# Supplementary material for: Long-term neuropsychiatric and neuropsychological impact of the pandemic in Italian COVID-19 family clusters, including children and parents
Source: PLoS One. 2025 Apr 24;20(4):e0321366. doi: 10.1371/journal.pone.0321366 (PMC12021208; doi:10.1371/journal.pone.0321366)
Supplement: Table S1 — (DOCX) [file pone.0321366.s001.docx]

*Table.S1 –* Neuropsychiatric assessment for children and their parents at the COVID-19 family cluster clinic (CovFC).

| ***Ages*** | ***n/N (response rate)*** | ***Neuropsychology evaluation*** | ***Child traumatic stress disorder evaluation*** | ***Emotional-behavior problems evaluation*** | ***Parents traumatic stress disorder evaluation*** | ***Depression, anxiety and stress evaluation*** |
| --- | --- | --- | --- | --- | --- | --- |
| ***1.5-5*** | 22/24 (91.7%) |  |  | Child Behavior Checklist (*CBCL)* |  |  |
| ***3-12*** | 57/61 (93.1%) |  | Trauma Symptom Checklist for Young Children (*TSCYC)* |  |  |  |
| ***3-12*** | 53/61 (86.9%) |  |  | Strengths and Difficulties Questionnaire (*SDQ)* |  |  |
| ***6-18*** | 47/53 (88.7%) |  |  | Child Behavior Checklist (*CBCL)* |  |  |
| ***3-18*** | 53/71 (74.7%) | Leiter International Performance Scale, third edition  *(Leiter-3)* |  |  |  |  |
| ***8-18*** | 39/44 (88.6%) |  | Trauma Symptom Checklist for Young Children (*TSCC)* |  |  |  |
| ***Parents*** | 66/81 (74.1%) |  |  |  | Impact of Event Scale Revised  *(IES-R)* |  |
| ***Parents*** | 73/81 (90.1%) |  |  |  |  | Depression Anxiety Stress Scale 21 *(DASS-21)* |
