# Supplementary material for: Long-term neuropsychiatric and neuropsychological impact of the pandemic in Italian COVID-19 family clusters, including children and parents
Source: PLoS One. 2025 Apr 24;20(4):e0321366. doi: 10.1371/journal.pone.0321366 (PMC12021208; doi:10.1371/journal.pone.0321366)
Supplement: Supplementary Questionnaire — (DOCX) [file pone.0321366.s002.docx]

*Supplementary Questionnaire -* Self-administered questionnaire assessing stress-related symptoms in children and parents.

Self- administered questionnaire

Please note: Other information may be retrieved and supplemented through anamnestic-clinical data collected by pediatricians

Name: …………………………………………….

Surname:…………………………………………..

Date of birth: …………………………………… M/F

Doctor/ psychologist (who fill)…………………………

Date of issue……………………………..

**(Filling in, writing or underlining- tick)**

**Family situation**

- Married parents
- Separated parents
- Deceased parent
- N° of brothers……..
- N° of sisters ………

**Score of SES …………**

**Eventual familiarity for neuropsychiatric conditions (who, whick problem)**

Describe

**History of previous trauma**

Describe

**Questions about COVID-19**

- Has anyone in the family tested positive for COVID-19?
- Has anyone been hospitalized?
- Has anyone into quarantine? Who? When? Describe
- Did your sleep change during home isolation ? (sleep more, less, same time) Describe

- Did your feeding change during home isolation? (eat more, less, same)

Describe

- Did your body weight change during home isolation?

Describe

- Did your time of use of devices change home isolation?

Describe

- Was there a variation of activities (educational/working routines, physical activity, and daily habits) during home isolation?

Describe

**Self-perceived stress-related symptoms (thinking about the pandemic period, mark any conditions among those listed below that affected you)**

**Children:**

- Physical stress symptoms

Headache, stomach pain, difficulty breathing, tachycardia, sweating of the hands, chest pain, agitation, sleep problems, fatigue, dizziness, loss of appetite, skin irritation, ringing in the ears.

- Behavioural stress symptoms:

Gnashing of teeth, compulsive feeding, critical attitude towards others, overbearing behaviour, difficulty in completing tasks.

- Emotional stress symptoms:

Tension, anger, restlessness, nervousness, anxiety, bad mood and frequent crying, sadness and unhappiness, demotivation, predisposition to agitation.

- Cognitive stress symptoms:

Difficulty in thinking clearly, problem making decisions, distraction/inattention, constant concern, loss of sense of humor, pessimism, confusion and mental turbulence, lack of creativity.

| **Father**  Physical stress symptoms:  Headache, stomach pain, difficulty breathing, tachycardia, sweating of the hands, chest pain, agitation, sleep problems, fatigue, dizziness, loss of appetite, skin irritation, ringing in the ears, hypertension and sexual problems.  Behavioural stress symptoms:  Gnashing of teeth, compulsive feeding, critical attitude towards others, overbearing behaviour, difficulty in completing tasks, frequent drinking and professional errors.  Emotional stress symptoms:  Tension, anger, restlessness, nervousness, anxiety, bad mood and frequent crying, sadness and unhappiness, demotivation, predisposition to agitation, sense of helplessness and predisposition to feeling upset.  Cognitive stress symptoms:  Difficulty in thinking clearly, problem making decisions, distraction/inattention, constant concern, loss of sense of humor, pessimism, confusion, mental turbulence, lack of creativity, difficulties in setting priorities. | **Mother**  Physical stress symptoms:  Headache, stomach pain, difficulty breathing, tachycardia, sweating of the hands, chest pain, agitation, sleep problems, fatigue, dizziness, loss of appetite, skin irritation, ringing in the ears, hypertension and sexual problems.  Behavioural stress symptoms:  Gnashing of teeth, compulsive feeding, critical attitude towards others, overbearing behaviour, difficulty in completing tasks, frequent drinking and professional errors.  Emotional stress symptoms:  Tension, anger, restlessness, nervousness, anxiety, bad mood and frequent crying, sadness and unhappiness, demotivation, predisposition to agitation, sense of helplessness and predisposition to feeling upset.  Cognitive stress symptoms:  Difficulty in thinking clearly, problem making decisions, distraction/inattention, constant concern, loss of sense of humor, pessimism, confusion, mental turbulence, lack of creativity, difficulties in setting priorities. |
| --- | --- |

**Anamnesis**

Stages of development- critical events- clinical data

……………………………………………………………………………………………………

……………………………………………………………………………………………………

……………………………………………………………………………………………………

**Anamnesis of neurodevelopmental pathologies**

……………………………………………………………………………………………………

……………………………………………………………………………………………………

……………………………………………………………………………………………………
