# Supplementary material for: Long-term neuropsychiatric and neuropsychological impact of the pandemic in Italian COVID-19 family clusters, including children and parents
Source: PLoS One. 2025 Apr 24;20(4):e0321366. doi: 10.1371/journal.pone.0321366 (PMC12021208; doi:10.1371/journal.pone.0321366)
Supplement: Table S2 — (DOCX) [file pone.0321366.s003.docx]

*Table.S2 –* Clinical cut-off for the Strengths and Difficulties Questionnaire (SDQ 4-17) subscales.

| Subscale | Subclinical cut-off | Clinical cut-off |
| --- | --- | --- |
| Emotional symptoms | >4 | >6 |
| Conduct problems | >4 | >5 |
| Hyperactivity-inattention problems | >6 | >8 |
| Peer problems | >3 | >5 |
| Prosociality behaviour symptoms | >4 | >3 |
