# Supplementary material for: Long-term neuropsychiatric and neuropsychological impact of the pandemic in Italian COVID-19 family clusters, including children and parents
Source: PLoS One. 2025 Apr 24;20(4):e0321366. doi: 10.1371/journal.pone.0321366 (PMC12021208; doi:10.1371/journal.pone.0321366)
Supplement: Table S3 — (DOCX) [file pone.0321366.s004.docx]

*Table.S3 –* Clinical cut-off for the Trauma Symptom Checklist for Young Children (TSCYC) and the Trauma Symptom Checklist for Children (TSCC) subscales.

| Subscale | Subclinical cut-off | Clinical cut-off |
| --- | --- | --- |
| TSCYC | >65 | >70 |
| TSCC |  |  |
| anxiety, depression, anger, post-traumatic stress, dissociation subscales | >60 | >65 |
| sexual concerns subscale | >65 | >70 |
