# Supplementary material for: Long-term neuropsychiatric and neuropsychological impact of the pandemic in Italian COVID-19 family clusters, including children and parents
Source: PLoS One. 2025 Apr 24;20(4):e0321366. doi: 10.1371/journal.pone.0321366 (PMC12021208; doi:10.1371/journal.pone.0321366)
Supplement: Table S4 — (DOCX) [file pone.0321366.s005.docx]

*Table.S4 -* Self-perceived stress-related symptoms among children (N=77) and parents (N=81) enrolled in the study.

|  | **Physical, N (%)** | | |  | **Behavioral, N (%)** | | |  | **Emotional, N (%)** | | |  | **Cognitive, N (%)** | | | | |  |
| --- | --- | --- | --- | --- | --- | --- | --- | --- | --- | --- | --- | --- | --- | --- | --- | --- | --- | --- |
|  | ***0*** | ***1-2*** | ***≥ 3*** | ***P-value*** | ***0*** | ***1*** | ***≥ 2*** | ***P-value*** | ***0*** | ***1-2*** | ***≥ 3*** | ***P-value*** | ***0*** | ***1-2*** | | | ***≥ 3*** | ***P-value*** |
| Overall  (N=158) | 71 (44.9) | 42 (26.6) | 45 (28.5) | - | 110 (69.6) | 34 (21.5) | 14 (8.9) | - | 46 (29.1) | 45 (24.5) | 67 (42.4) | - | 85 (53.8) | 43 (27.2) | | | 30 (20) | - |
| Self-perceived stress-related symptoms, overall |  |  |  |  |  |  |  |  |  |  |  |  |  |  | | |  |  |
| No  (N=22) | 22 (100) | 0 (0) | 0 (0) | **< .01** | 22 (100) | 0 (0) | 0 (0) | **< .01** | 22 (100) | 0 | 0 | **< .0001** | 22 (100) | 0 (0) | | | 0 (0) | **< .01** |
| Yes  (N=136) | 49 (36) | 42 (30.9) | 45 (33.1) |  | 88 (64.7) | 34 (25) | 14 (10.3) |  | 24 (17.7) | 45 (33) | 67 (49.3) |  | 63 (46.3) | 43 (31.6) | | | 30 (22.1) |  |
| Self-perceived stress-related symptoms, physics |  |  |  |  |  |  |  |  |  |  |  |  |  |  | | | |  |
| No  (N=71) | 71 (98.6) | 0 (0) | 0 (0) | **<.01** | 65 (91.6) | 5 (7) | 1 (1.4) | **<.01** | 27 (38) | 27 (38) | 17 (23.9) | **<.01** | 50 (70.42) | 15 (21.13) | | | 6 (8.45) | **< .01** |
| Yes  (N=87) | 0 (0) | 42 (48.3) | 45 (52.7) |  | 45 (51.7) | 29 (33.3) | 13 (14.9) |  | 19 (21.8) | 18 (20.7) | 50 (57.5) |  | 35 (40.2) | 28 (32.2) | | | 24 (27.6) |  |
| Self-perceived stress-related symptoms, behavioral |  |  |  |  |  |  |  |  |  |  |  |  |  |  | | |  |  |
| No  (N=110) | 65 (59.1) | 27 (24.6) | 18 (16.4) | **< .01** | 110 (100) | 0 (0) | 0 (0) | **< .01** | 41 (37.3) | 34 (30.9) | 35 (31.8) | **< .01** | 75 (68.2) | 28 (25.5) | | | 7 (6.4) | **< .01** |
| Yes  (N=48) | 6 (12.5) | 15 (31.3) | 27 (56.3) |  | 0 (0) | 34 (70.8) | 14 (29.2) |  | 5 (10.4) | 11 (22.9) | 32 (66.7) |  | 10 (20.8) | 15 (31.3) | | | 23 (47.9) |  |
| Self-perceived stress-related symptoms, emotional |  | | |  |  | | |  |  | | |  |  | | | | |  |
| No  (N=46) | 27 (58.7) | 15 (32.6) | 4 (8.7) | **<.01** | 41 (89.1) | 5 (10.9) | 0 (0) | **<.01** | 46 (100) | 0 (0) | 0 (0) | **<.01** | 35 (76.1) | 10 (21.7) | | | 1 (2.2) | **<.01** |
| Yes  (N=112) | 44 (39.3) | 27 (24.1) | 41 (36.6) |  | 69 (61.6) | 29 (25.9) | 14 (12.5) |  | 0 (0) | 45 (40.2) | 67 (59.8) |  | 50 (44.6) | 33 (29.5) | | | 29 (25.9) |  |
| Self-perceived stress-related symptoms, cognitive |  |  |  |  |  |  |  |  |  |  |  |  |  |  | | |  |  |
| No  (N=85) | 50 (58.8) | 22 (25.9) | 13 (15.3) | **<.01** | 75 (88.2) | 8 (9.4) | 2 (2.4) | **< .01** | 35 (41.2) | 26 (30.6) | 24 (28.2) | **<.01** | 85 (100) | 0 (0) | | 0 (0) | | **<.01** |
| Yes  (N=73) | 21 (28.8) | 20 (27.4) | 32 (43.8) |  | 35 (48) | 26 (35.6) | 12 (16.4) |  | 11 (15.1) | 19 (26.0) | 43 (58.9) |  | 0 (0) | 43 (58.9) | | | 30 (41.1) |  |
|  |  |  |  |  |  |  |  |  |  |  |  |  |  |  | | |  |  |
| Pandemic wave |  |  |  |  |  |  |  |  |  |  |  |  |  |  | | |  |  |
| Parental  (N=39) | 34 (87.2) | 4 (10.3) | 1 (2.6) | **<.01** | 36 (92.3) | 3 (7.7) | 0 (0) | **.01** | 14 (35.9) | 16 (41) | 9 (23.1) | **<.01** | 26 (66.7) | 12 (30.8) | 1 (2.6) | | |  |
| Delta  (N=59) | 22 (37.9) | 16 (27.6) | 20 (34.5) |  | 37 (63.8) | 14 (24.1) | 7 (12.1) |  | 9 (15.5) | 20 (34.5) | 29 (50) |  | 30 (51.7) | 13 (22.4) | | | 15 (25.9) | **.04** |
| Omicron  (N=61) | 15 (24.6) | 22 (36.1) | 24 (39.3) |  | 37 (60.7) | 17 (27.9) | 7 (11.5) |  | 23 (37.7) | 9 (14.7) | 29 (47.5) |  | 29 (47.5) | 18 (29.5) | | | 14 (23) |  |
| COVID-19 symptoms |  |  |  |  |  |  |  |  |  |  |  |  |  |  | | |  |  |
| Asymptomatic  (N=45) | 31 (68.9) | 9 (20) | 5 (11.1) | **<.01** | 37 (82.2) | 5 (11.1) | 3 (6.7) | .08 | 16 (35.6) | 18 (40) | 11 (24.4) | **.01** | 28 (62.2) | 13 (28.9) | | | 4 (8.9) | .12 |
| Symptomatic  (N=113) | 40 (35.4) | 33 (29.2) | 40 (35.4) |  | 73 (64.6) | 29 (25.7) | 11 (9.7) |  | 30 (26.6) | 27 (23.9) | 56 (49.6) |  | 57 (50.4) | 30 (26.6) | | | 26 (23) |  |
