# Supplementary material for: Long-term neuropsychiatric and neuropsychological impact of the pandemic in Italian COVID-19 family clusters, including children and parents
Source: PLoS One. 2025 Apr 24;20(4):e0321366. doi: 10.1371/journal.pone.0321366 (PMC12021208; doi:10.1371/journal.pone.0321366)
Supplement: Table S5 — (DOCX) [file pone.0321366.s006.docx]

*Table.S5-* Assessment of emotional-behavioral and PTSD-related symptoms in children through the CBCL, SDQ-4-17, TSCYC, and TSCC questionnaires and the Leiter-3 test.

|  |  | **Self-perceived stress-related symptoms, overall** | | | **Self-perceived stress-related symptoms, physics** | | | **Self-perceived stress-related symptoms, behavioral** | | | **Self-perceived stress-related symptoms, emotional** | | | **Self-perceived stress-related symptoms, cognitive** | | |
| --- | --- | --- | --- | --- | --- | --- | --- | --- | --- | --- | --- | --- | --- | --- | --- | --- |
|  | ***Response rate*** | ***No*** | ***Yes*** | ***P-value*** | ***No*** | ***Yes*** | ***P-value*** | ***No*** | ***Yes*** | ***P-value*** | ***No*** | ***Yes*** | ***P-value*** | ***No*** | ***Yes*** | ***P-value*** |
| Children |  | ***N=11*** | ***N=66*** |  | ***N=34*** | ***N=43*** |  | ***N=53*** | ***N=24*** |  | ***N=21*** | ***N=56*** |  | ***N=42*** | ***N=35*** |  |
| CBCL, children aged 1.5-5 years | 22/24 (91.7) |  |  |  |  |  |  |  |  |  |  |  |  |  |  |  |
| Internalizing problems, N (%) |  |  |  |  |  |  |  |  |  |  |  |  |  |  |  |  |
| <65  (N=22) |  | 2 (100) | 20 (100) | - | 12 (100) | 10 (100) | - | 19 (100) | 3 (100) | - | 3 (100) | 19 (100) | - | 19 (100) | 3 (100) | - |
| ≥ 65  (N=0) |  | 0 (0) | 0 (0) |  | 0 (0) | 0 (0) |  | 0 (0) | 0 (0) |  | 0 (0) | 0 (0) |  | 0 (0) | 0 (0) |  |
| Externalizing problems |  |  |  |  |  |  |  |  |  |  |  |  |  |  |  |  |
| <65  (N=22) |  | 2 (100) | 20 (100) | - | 12 (100) | 10 (100) | - | 19 (100) | 3 (100) | - | 3 (100) | 19 (100) | - | 19 (100) | 3 (100) | - |
| ≥ 65  (N=0) |  | 0 (0) | 0 (0) |  | 0 (0) | 0 (0) |  | 0 (0) | 0 (0) |  | 0 (0) | 0 (0) |  | 0 (0) | 0 (0) |  |
| Total problems |  |  |  |  |  |  |  |  |  |  |  |  |  |  |  |  |
| <65  (N=22) |  | 2 (100) | 20 (100) | - | 12 (100) | 10 (100) | - | 19 (100) | 3 (100) | - | 3 (100) | 19 (100) | - | 19 (100) | 3 (100) | - |
| ≥ 65  (N=0) |  | 0 (0) | 0 (0) |  | 0 (0) | 0 (0) |  | 0 (0) | 0 (0) |  | 0 (0) | 0 (0) |  | 0 (0) | 0 (0) |  |
| CBCL, children aged 6-18 years | 47/53 (88.7) |  |  |  |  |  |  |  |  |  |  |  |  |  |  |  |
| Internalizing problems |  |  |  |  |  |  |  |  |  |  |  |  |  |  |  |  |
| <65  (N=31) |  | 4 (100) | 27 (62.8) | .18 | 13 (86.7) | 18 (56.2) | **.04** | 22 (81.5) | 9 (45) | **.01** | 10 (90.9) | 21 (58.3) | **.04** | 14 (87.5) | 17 (54.8) | **.03** |
| ≥ 65  (N=16) |  | 0 (0) | 16 (37.2) |  | 2 (13.3) | 14 (43.8) |  | 5 (18.5) | 11 (55) |  | 1 (9.1) | 15 (41.7) |  | 2 (12.5) | 14 (45.2) |  |
| Externalizing problems |  |  |  |  |  |  |  |  |  |  |  |  |  |  |  |  |
| <65  (N=45) |  | 4 (100) | 41 (95.4) | .84 | 14 (93.3) | 31 (96.9) | .44 | 26 (96.3) | 19 (95) | .50 | 11 (100) | 34 (94.4) | .58 | 15 (93.8) | 30 (96.8) | .46 |
| ≥ 65  (N=2) |  | 0 (0) | 2 (4.6) |  | 1 (6.7) | 1 (3.1) |  | 1 (3.7) | 1 (5) |  | 0 (0) | 2 (5.6) |  | 1 (6.2) | 1 (3.2) |  |
| Total problems |  |  |  |  |  |  |  |  |  |  |  |  |  |  |  |  |
| <65  (N=40) |  | 4 (100) | 36 (83.7) | .51 | 13 (86.7) | 27 (84.4) | .34 | 24 (88.9) | 16 (80) | .23 | 11 (100) | 29 (80.6) | .13 | 13 (81.3) | 27 (87.1) | .28 |
| ≥ 65  (N=7) |  | 0 (0) | 7 (16.3) |  | 2 (13.3) | 5 (15.6) |  | 3 (11.1) | 4 (20) |  | 0 (0) | 7 (19.4) |  | 3 (18.7) | 4 (12.9) |  |
| SDQ | 53/61 (86.9) |  |  |  |  |  |  |  |  |  |  |  |  |  |  |  |
| 0-13  (N=45) |  | 4 (100) | 41 (83.7) | .51 | 21 (95.4) | 24 (77.4) | .05 | 31 (96.9) | 14 (66.7) | **<.01** | 11(91.7) | 34 (82.9) | .23 | 26 (100) | 19 (70.4) | **<.01** |
| 14-16  (N=6) |  | 0 (0) | 6 (12.2) |  | 1 (4.6) | 5 (16.1) |  | 1 (3.1) | 5 (23.8) |  | 1 (8.3) | 5 (12.2) |  | 0 (0) | 6 (22.2) |  |
| ≥18  (N=2) |  | 0 (0) | 2 (4.1) |  | 0 (0) | 2 (6.5) |  | 0 (0) | 2 (9.5) |  | 0 (0) | 2 (4.9) |  | 0 (0) | 2 (7.4) |  |
| TSCYC | 57/61 (93.1) |  |  |  |  |  |  |  |  |  |  |  |  |  |  |  |
| <60  (N=47) |  | 3 (75) | 43 (81.1) | .35 | 20 (87) | 26 (76.5) | .09 | 31 (86.1) | 15 (71.4) | .06 | 10 (76.9) | 36 (81.8) | .12 | 25 (83.4) | 21 (77.8) | .12 |
| 60-64  (N=2) |  | 0 (0) | 2 (3.8) |  | 0 (0) | 2 (5.9) |  | 1 (2.8) | 1 (4.8) |  | 1 (7.7) | 1 (2.3) |  | 1 (3.3) | 1 (3.7) |  |
| ≥ 64  (N=9) |  | 1 (25) | 8 (15.1) |  | 3 (13) | 6 (17.6) |  | 4 (11.1) | 5 (23.8) |  | 2 (15.4) | 7 (15.9) |  | 4 (13.3) | 5 (18.5) |  |
| TSCC | 39/44  (88.6) |  |  |  |  |  |  |  |  |  |  |  |  |  |  |  |
| <60  (N=36) |  | 4 (100) | 32 (91.4) | .72 | 11 (91.7) | 25 (92.6) | .31 | 22 (95.7) | 14 (87.6) | .21 | 12 (100) | 24 (88.9) | .32 | 10 (83.3) | 26 (96.3) | .07 |
| 60-64  (N=2) |  | 0 (0) | 2 (5.7) |  | 1 (8.3) | 1 (3.7) |  | 1 (4.3) | 1 (6.2) |  | 0 (0) | 2 (7.4) |  | 2 (16.7) | 0 (0) |  |
| ≥ 64  (N=1) |  | 0 (0) | 1 (2.9) |  | 0 (0) | 1 (3.7) |  | 0 (0) | 1 (6.2) |  | 0 (0) | 1 (3.7) |  | 0 (0) | 1 (3.7) |  |
| Leiter non verbal memory | 53/71 (74.7) |  |  |  |  |  |  |  |  |  |  |  |  |  |  |  |
| <90  (N=13) |  | 3 (50) | 10 (21.3) | .06 | 7 (33.3) | 6 (18.7) | .45 | 8 (24.2) | 5 (25) | 1 | 7 (53.8) | 6 (15) | **<.01** | 5 (19.2) | 8 (29.7) | .45 |
| 90-109  (N=16) |  | 1 (16.7) | 15 (31.9) |  | 5 (23.8) | 11 (34.4) |  | 10 (30.3) | 6 (30) |  | 3 (23.1) | 13 (32.5) |  | 7 (26.9) | 9 (33.3) |  |
| >109  (N=24) |  | 2 (33.3) | 22 (46.8) |  | 9 (42.9) | 15 (46.9) |  | 15 (45.5) | 9 (45) |  | 3 (23.1) | 21 (52.5) |  | 14 (53.9) | 10 (37) |  |
| Leiter process speed | 53/71 (74.7) |  |  |  |  |  |  |  |  |  |  |  |  |  |  |  |
| <90  (N=23) |  | 2 (33.3) | 21 (44.7) | .10 | 11 (52.4) | 12 (37.5) | .06 | 16 (48.5) | 7 (35) | .59 | 6 (46.1) | 17 (42.5) | **.03** | 13 (50) | 10 (37) | .05 |
| 90-109  (N=27) |  | 3 (50) | 24 (51.1) |  | 9 (42.9) | 18 (56.3) |  | 15 (45.4) | 12 (60) |  | 5 (38.5) | 22 (55) |  | 11 (42.3) | 16 (59.3) |  |
| >109  (N=3) |  | 1 (16.7) | 2 (4.2) |  | 1 (4.7) | 2 (6.2) |  | 2 (6.1) | 1 (5) |  | 2 (15.4) | 1 (2.5) |  | 2 (7.7) | 1 (3.7) |  |
