# Supplementary material for: Long-term neuropsychiatric and neuropsychological impact of the pandemic in Italian COVID-19 family clusters, including children and parents
Source: PLoS One. 2025 Apr 24;20(4):e0321366. doi: 10.1371/journal.pone.0321366 (PMC12021208; doi:10.1371/journal.pone.0321366)
Supplement: Table S6 — (DOCX) [file pone.0321366.s007.docx]

*Table.S6*- Emotional-behavioral and PTSD-related symptoms in children according to socio-demographic factors, assessed with the CBCL, SDQ-4-17, TSCYC, and TSCC questionnaires.

|  | | **Sex** | | | **Non-psychiatric underlying conditions** | | | **Familiarity for neuropsychiatric condition** | | | **SES** | | |
| --- | --- | --- | --- | --- | --- | --- | --- | --- | --- | --- | --- | --- | --- |
|  | ***Response rate*** | ***Male*** | ***Female*** | ***P-value*** | ***No*** | ***Yes*** | ***P-value*** | ***No*** | ***Yes*** | ***P-value*** | ***Medium - low*** | ***Medium - high*** | ***P-value*** |
|  |  | 44 (57.1) | 33 (42.9) | **-** | 54 (70.1) | 23 (29.9) | - | 40 (52) | 37 (48) |  | 36 (48) | 39 (52) |  |
| CBCL, children aged 1.5-5 years | 22/24 (91.7) |  |  |  |  |  |  |  |  |  |  |  |  |
| Internalizing problems, N (%) |  |  |  |  |  |  |  |  |  |  |  |  |  |
| <65  (N=22) |  | 12 (100) | 10 (100) | - | 18 (100) | 4 (100) | - | 8 (100) | 14 (100) |  | 12 (100) | 9 (100) | - |
| ≥ 65  (N=0) |  | 0 (0) | 0 (0) |  | 0 (0) | 0 (0) |  | 0 (0) | 0 (0) |  | 0 (0) | 0 (0) |  |
| Externalizing problems |  |  |  |  |  |  |  |  |  |  |  |  |  |
| <65  (N=22) |  | 12 (100) | 10 (100) | - | 18 (100) | 4 (100) | - | 8 (100) | 14 (100) |  | 12 (100) | 9 (100) | - |
| ≥ 65  (N=0) |  | 0 (0) | 0 (0) |  | 0 (0) | 0 (0) |  | 0 (0) | 0 (0) |  | 0 (0) | 0 (0) |  |
| Total problems |  |  |  |  |  |  |  |  |  |  |  |  |  |
| <65  (N=22) |  | 12 (100) | 10 (100) | - |  | 4 (100) | - | 8 (100) | 14 (100) |  | 12 (100) | 9 (100) | - |
| ≥ 65  (N=0) |  | 0 (0) | 0 (0) |  | 0 (0) | 0 (0) |  | 0 (0) | 0 (0) |  | 0 (0) | 0 (0) |  |
| CBCL, children aged 6-18 years | 47/53 (88.7) |  |  |  |  |  |  |  |  |  |  |  |  |
| Internalizing problems |  |  |  |  |  |  |  |  |  |  |  |  |  |
| <65  (N=31) |  | 19 (67.9) | 12 (63.2) | .74 | 23 (76.7) | 8 (47.1) | **.04** | 17 (68) | 14 (63.6) |  | 11 (50) | 20 (83.3) | **.02** |
| ≥ 65  (N=16) |  | 9 (32.1) | 7 (36.8) |  | 7 (23.3) | 9 (52.9) |  | 8 (32) | 8 (36.4) |  | 11 (50) | 4 (16.7) |  |
| Externalizing problems |  |  |  |  |  |  |  |  |  |  |  |  |  |
| <65  (N=45) |  | 27 (96.4) | 18 (97.7) | .49 | 28 (93.3) | 17 (100) | **.40** | 24 (96) | 21 (95.4) |  | 22 (100) | 23 (95.8) | .52 |
| ≥ 65  (N=2) |  | 1 (3.6) | 1 (5.3) |  | 2 (6.7) | 0 (0) |  | 1 (4) | 1 (4.5) |  | 0 (0) | 1 (4.2) |  |
| Total problems |  |  |  |  |  |  |  |  |  |  |  |  |  |
| <65  (N=40) |  | 23 (82.1) | 17 (89.5) | .27 | 27 (90) | 13 (76.5) | .15 | 23 (92) | 17 (77.3) |  | 18 (81.8) | 22 (91.7) | .22 |
| ≥ 65  (N=7) |  | 5 (17.9) | 2 (10.5) |  | 3 (10) | 4 (23.5) |  | 2 (8) | 5 (22.7) |  | 4 (18.2) | 2 (8.3) |  |
| SDQ | 53/61 (86.9) |  |  |  |  |  |  |  |  |  |  |  |  |
| 0-13  (N=45) |  | 28 (87.5) | 17 (81) | .14 | 32 (86.5) | 13 (81.2) | .15 | 27 (87.1) | 18 (81.8) |  | 22 (84.6) | 22 (88) | .17 |
| 14-16  (N=6) |  | 3 (9.4) | 3 (14.2) |  | 4 (10.8) | 2 (12.5) |  | 3 (9.7) | 3 (13.6) |  | 3 (11.5) | 2 (8) |  |
| ≥18  (N=2) |  | 1 (3.1) | 1 (4.8) |  | 1 (2.7) | 1 (6.2) |  | 1 (3.2) | 1 (4.5) |  | 1 (3.9) | 1 (4) |  |
| TSCYC | 57/61 (93.1) |  |  |  |  |  |  |  |  |  |  |  |  |
| <60  (N=47) |  | 25 (78.1) | 21 (84) | .09 | 34 (85) | 12 (70.6) | **.03** | 29 (90.6) | 17 (68) |  | 24 (82.8) | 21 (80.8) | .15 |
| 60-64  (N=2) |  | 2 (6.3) | 0 (0) |  | 2 (5) | 0 (0) |  | 0 (0) | 2 (8) |  | 1 (3.4) | 1 (3.8) |  |
| ≥ 64  (N=9) |  | 5 (15.6) | 4 (16) |  | 4 (10) | 5 (29.4) |  | 3 (9.4) | 6 (24) |  | 4 (13.8) | 4 (15.4) |  |
| TSCC | 39/44  (88.6) |  |  |  |  |  |  |  |  |  |  |  |  |
| <60  (N=36) |  | 21 (91.3) | 15 (93.7) | .15 | 20 (87) | 16 (100) | .19 | 20 (90.9) | 16 (94.1) |  | 18 (90) | 18 (94.7) | .26 |
| 60-64  (N=2) |  | 2 (8.7) | 0 (0) |  | 2 (8.7) | 0 (0) |  | 1 (4.5) | 1 (6) |  | 1 (5) | 1 (5.3) |  |
| ≥ 64  (N=1) |  | 0 (0) | 1 (6.3) |  | 1 (4.3) | 0 (0) |  | 1 (4.5) | 0 (0) |  | 1 (5) | 0 (0) |  |
| Leiter non verbal memory | 53/71 (74.7) |  |  |  |  |  |  |  |  |  |  |  |  |
| <90  (N=13) |  | 6 (18.2) | 7 (35) | .34 | 11 (31.4) | 2 (11.1) | .07 | 7 (26.9) | 6 (22.3) |  | 7 (30.4) | 5 (17.9) | .16 |
| 90-109  (N=16) |  | 10 (30.3) | 6 (30) |  | 12 (34.3) | 4 (22.2) |  | 7 (26.9) | 9 (33.3) |  | 9 (39.1) | 7 (25) |  |
| >109  (N=24) |  | 17 (51.5) | 7 (35) |  | 12 (34.3) | 12 (66.7) |  | 12 (46.1) | 12 (44.4) |  | 7 (30.4) | 16 (57.1) |  |
| Leiter process speed | 53/71 (74.7) |  |  |  |  |  |  |  |  |  |  |  |  |
| <90  (N=23) |  | 15 (45.5) | 8 (40) | **.04** | 17 (48.6) | 6 (33.3) | .06 | 9 (34.6) | 14 (51.9) |  | 11 (47.8) | 12 (42.9) | **.04** |
| 90-109  (N=27) |  | 15 (45.5) | 12 (60) |  | 16 (45.7) | 11 (61.1) |  | 15 (57.7) | 12 (44.4) |  | 12 (52.2) | 13 (46.4) |  |
| >109  (N=3) |  | 3 (9) | 0 (0) |  | 2 (5.7) | 1 (5.6) |  | 2 (7.7) | 1 (3.7) |  | 0 (0) | 3 (10.7) |  |
