# Supplementary material for: Long-term neuropsychiatric and neuropsychological impact of the pandemic in Italian COVID-19 family clusters, including children and parents
Source: PLoS One. 2025 Apr 24;20(4):e0321366. doi: 10.1371/journal.pone.0321366 (PMC12021208; doi:10.1371/journal.pone.0321366)
Supplement: Table S7 — (DOCX) [file pone.0321366.s008.docx]

*Table.S7*- Emotional-behavioral and PTSD-related symptoms in children according to COVID-19-related factors, assessed with the CBCL, SDQ-4-17, TSCYC, and TSCC questionnaires.

|  | | **COVID-19** | | | **COVID-19 symptoms** | | | **Pandemic wave** | | | | **Duration of home isolation** | | |
| --- | --- | --- | --- | --- | --- | --- | --- | --- | --- | --- | --- | --- | --- | --- |
|  | ***Children*** | ***No*** | ***Yes*** | ***P-value*** | ***Asymptomatic*** | ***Symptomatic*** | ***P-value*** | ***Parental*** | ***Delta*** | ***Omicron*** | ***P-value*** | ***<3*** | ***≥ 3*** | ***P-value*** |
|  |  | 12 (15.6) | 65 (84.4) | - | 20 (26) | 57 (74) | - | 19 (24.7) | 32 (41.5) | 26 (33.8) |  | 31 (40.3) | 46 (59.7) | - |
| CBCL, children aged 1.5-5 years | 22/24 (91.7) |  |  |  |  |  |  |  |  |  |  |  |  |  |
| Internalizing problems, N (%) |  |  |  |  |  |  |  |  |  |  |  |  |  |  |
| <65  (N=22) |  | 6 (100) | 16 (100) | - | 1 (100) | 16 (100) | - | 6 (100) | 9 (100) | 7 (100) | - | 5 (100) | 17 (100) | - |
| ≥ 65  (N=0) |  | 0 (0) | 0 (0) |  | 0 (0) | 0 (0) |  | 0 (0) | 0 (0) | 0 (0) |  | 0 (0) | 0 (0) |  |
| Externalizing problems |  |  |  |  |  |  |  |  |  |  |  |  |  |  |
| <65  (N=22) |  | 6 (100) | 16 (100) | - | 1 (100) | 16 (100) | - | 6 (100) | 9 (100) | 7 (100) | - | 5 (100) | 17 (100) | - |
| ≥ 65  (N=0) |  | 0 (0) | 0 (0) |  | 0 (0) | 0 (0) |  | 0 (0) | 0 (0) | 0 (0) |  | 0 (0) | 0 (0) |  |
| Total problems |  |  |  |  |  |  |  |  |  |  |  |  |  |  |
| <65  (N=22) |  | 6 (100) | 16 (100) | - | 1 (100) | 16 (100) | - | 6 (100) | 9 (100) | 7 (100) | - | 5 (100) | 17 (100) | - |
| ≥ 65  (N=0) |  | 0 (0) | 0 (0) |  | 0 (0) | 0 (0) |  | 0 (0) | 0 (0) | 0 (0) |  | 0 (0) | 0 (0) |  |
| CBCL, children aged 6-18 years | 47/53 (88.7) |  |  |  |  |  |  |  |  |  |  |  |  |  |
| Internalizing problems |  |  |  |  |  |  |  |  |  |  |  |  |  |  |
| <65  (N=31) |  | 4 (100) | 27 (62.8) | .18 | 4 (80) | 23 (60.5) | .29 | 8 (100) | 14 (77.8) | 9 (42.9) | **<.01** | 13 (59.1) | 18 (72) | .35 |
| ≥ 65  (N=16) |  | 0 (0) | 16 (37.2) |  | 1 (20) | 15 (39.5) |  | 0 (0) | 4 (22.2) | 12 (57.1) |  | 9 (40.9) | 7 (28) |  |
| Externalizing problems |  |  |  |  |  |  |  |  |  |  |  |  |  |  |
| <65  (N=45) |  | 3 (75) | 42 (97.7) | .16 | 5 (100) | 37 (97.4) | .88 | 7 (87.5) | 18 (100) | 20 (95.2) | .16 | 21 (95.5) | 24 (96) | .51 |
| ≥ 65  (N=2) |  | 1 (25) | 1 (2.3) |  | 0 (0) | 1 (2.6) |  | 1 (12.5) | 0 (0) | 1 (4.8) |  | 1 (4.5) | 1 (4) |  |
| Total problems |  |  |  |  |  |  |  |  |  |  |  |  |  |  |
| <65  (N=40) |  | 3 (75) | 37 (86.1) | .39 | 5 (100) | 32 (84.2) | .45 | 7 (87.5) | 16 (88.9) | 17 (81) | .12 | 19 (86.4) | 21 (84) | .31 |
| ≥ 65  (N=7) |  | 1 (25) | 6 (13.9) |  | 0 (0) | 6 (15.8) |  | 1 (12.5) | 2 (11.1) | 4 (19) |  | 3 (13.6) | 4 (16) |  |
| SDQ | 53/61 (86.9) |  |  |  |  |  |  |  |  |  |  |  |  |  |
| 0-13  (N=45) |  | 7 (100) | 38 (82.6) | .29 | 5 (100) | 33 (80.5) | .37 | 11 (100) | 19 (86.4) | 15 (75) | **.01** | 18 (78.3) | 27 (90) | .08 |
| 14-16  (N=6) |  | 0 (0) | 6 (13) |  | 0 (0) | 6 (14.6) |  | 0 (0) | 2 (9.1) | 4 (20) |  | 4 (17.4) | 2 (6.7) |  |
| ≥18  (N=2) |  | 0 (0) | 2 (4.4) |  | 0 (0) | 2 (4.9) |  | 0 (0) | 1 (4.5) | 1 (5) |  | 1 (4.3) | 1 (3.3) |  |
| TSCYC | 57/61 (93.1) |  |  |  |  |  |  |  |  |  |  |  |  |  |
| <60  (N=47) |  | 7 (100) | 39 (78) | .20 | 5 (83.3) | 34 (77.3) | .33 | 11 (91.7) | 18 (81.8) | 17 (73.9) | **.02** | 20 (80) | 26 (81.3) | .05 |
| 60-64  (N=2) |  | 0 (0) | 2 (4) |  | 0 (0) | 2 (4.6) |  | 0 (0) | 1 (4.6) | 1 (4.4) |  | 2 (8) | 0 (0) |  |
| ≥ 64  (N=9) |  | 0 (0) | 9 (18) |  | 1 (16.7) | 8 (18.2) |  | 1 (8.3) | 3 (13.6) | 5 (21.7) |  | 3 (12) | 6 (18.7) |  |
| TSCC | 39/44  (88.6) |  |  |  |  |  |  |  |  |  |  |  |  |  |
| <60  (N=36) |  | 4 (100) | 32 (91.4) | .72 | 5 (83.3) | 27 (93) | .25 | 5 (100) | 15 (93.8) | 16 (88.8) | .18 | 17 (85) | 19 (100) | .12 |
| 60-64  (N=2) |  | 0 (0) | 2 (5.7) |  | 1 (16.7) | 1 (3.5) |  | 0 (0) | 1 (6.2) | 1 (5.6) |  | 2 (10) | 0 (0) |  |
| ≥ 64  (N=1) |  | 0 (0) | 1 (2.9) |  | 0 (0) | 1 (3.5) |  | 0 (0) | 0 (0) | 1 (5.6) |  | 1 (5) | 0 (0) |  |
| Leiter non verbal memory | 53/71 (74.7) |  |  |  |  |  |  |  |  |  |  |  |  |  |
| <90  (N=13) |  | 0 (0) | 13 (28.3) | **.04** | 3 (37.5) | 10 (26.3) | .07 | 3 (30) | 4 (18.2) | 6 (28.6) | **<.01** | 7 (29.2) | 6 (20.7) | .77 |
| 90-109  (N=16) |  | 3 (42.9) | 13 (28.3) |  | 1 (12.5) | 12 (31.6) |  | 2 (20) | 6 (27.3) | 8 (38.1) |  | 7 (29.2) | 9 (31) |  |
| >109  (N=24) |  | 4 (57.1) | 20 (43.4) |  | 4 (50) | 16 (42.1) |  | 5 (50) | 12 (54.5) | 7 (33.3) |  | 10 (41.6) | 14 (48.3) |  |
| Leiter process speed | 53/71 (74.7) |  |  |  |  |  |  |  |  |  |  |  |  |  |
| <90  (N=23) |  | 3 (42.9) | 20 (43.4) | .10 | 2 (25) | 18 (47.4) | .10 | 5 (50) | 9 (40.9) | 9 (42.9) | **.01** | 6 (24) | 17 (58.6) | **<.01** |
| 90-109  (N=27) |  | 3 (42.9) | 24 (52.2) |  | 6 (75) | 18 (47.4) |  | 5 (50) | 11 (50) | 11 (52.4) |  | 17 (70.8) | 10 (34.5) |  |
| >109  (N=3) |  | 1 (14.2) | 2 (4.4) |  | 0 (0) | 2 (5.3) |  | 0 (0) | 2 (9.1) | 1 (4.8) |  | 1 (4.2) | 2 (6.9) |  |
