# Supplementary material for: Long-term neuropsychiatric and neuropsychological impact of the pandemic in Italian COVID-19 family clusters, including children and parents
Source: PLoS One. 2025 Apr 24;20(4):e0321366. doi: 10.1371/journal.pone.0321366 (PMC12021208; doi:10.1371/journal.pone.0321366)
Supplement: Table S8 — (DOCX) [file pone.0321366.s009.docx]

*Table.S8 -* Neuropsychiatric and neuropsychological assessment of parents.

|  | **IES (66/81 = 74.1)** | | | **DASS 21 (73/81 = 90.1)** | | | | | | | | | | |
| --- | --- | --- | --- | --- | --- | --- | --- | --- | --- | --- | --- | --- | --- | --- |
|  |  |  |  | ***Depression*** | |  | ***Anxiety*** | | |  | ***Stress*** | | |  |
|  | **<1.1** | ***≥1.1*** | ***P-value*** | ***≤ 9*** | ***>9*** | ***P-value*** | ***≤ 7*** | ***>7*** | | ***P-value*** | ***≤14*** | ***>14*** | | ***P-value*** |
| Overall | 58 | 8 |  | 64 | 9 |  | 65 | 8 | |  | 57 | 16 | |  |
|  |  |  |  |  |  |  |  |  | |  |  |  | |  |
| Self-perceived stress-related symptoms, overall |  |  |  |  |  |  |  |  | |  |  |  | |  |
| No  (N=11) | 9 (100) | 0 (0) | .29 | 9 (100) | 0 (0) | .28 | 7 (77.8) | 2 (22.2) | | .20 | 9 (100) | 0 (0) | | .09 |
| Yes  (N=70) | 49 (86) | 8 (14) |  | 55 (85.9) | 9 (14.1) |  | 58 (90.6) | 6 (9.4) | |  | 48 (75) | 16 (25) | |  |
|  |  |  |  |  |  |  |  |  | |  |  |  | |  |
| Self-perceived stress-related symptoms, physics |  |  |  |  |  |  |  |  | |  |  |  | |  |
| No  (N=37) | 28 (96.6) | 1 (3.4) | **.04** | 33 (97.1) | 1 (2.9) | **.02** | 32 (94.1) | 2 (5.9) | | .12 | 29 (82.3) | 5 (14.7) | | .34 |
| Yes  (N=44) | 30 (81.1) | 7 (18.9) |  | 31 (79) | 8 (21) |  | 33 (84.6) | 6 (15.4) | |  | 28 (71.8) | 11 (28.2) | |  |
|  |  |  |  |  |  |  |  |  | |  |  |  | |  |
| Self-perceived stress-related symptoms, behavioral |  |  |  |  |  |  |  |  | |  |  |  | |  |
| No  (N=57) | 43 (95.6) | 2 (4.4) | **<.01** | 48 (92.3) | 4 (7.7) | .06 | 48 (92.3) | 4 (7.7) | | .12 | 42 (80.8) | 10 (19.2) | | .16 |
| Yes  (N=24) | 15 (71.4) | 6 (28.6) |  | 16 (76.2) | 5 (23.8) |  | 17 (81) | 4 (19) | |  | 15 (71.4) | 6 (28.6) | |  |
|  |  | |  |  | |  |  | | |  |  | | |  |
| Self-perceived stress-related symptoms, emotional |  |  |  |  | |  |  | |  |  |  | | |  |
| No  (N=25) | 19 (100) | 0 (0) | .05 | 18 (94.7) | 1 (5.3) | .20 | 17 (89.5) | 2 (10.5) | | .66 | 17 (89.5) | | 2 (10.5) | .11 |
| Yes  (N=56) | 39 (83) | 8 (17) |  | 46 (85.2) | 8 (14.8) |  | 48 (88.9) | 6 (11.1) | |  | 40 (74.1) | | 14 (25.9) |  |
|  |  |  |  |  |  |  |  | | |  |  | |  |  |
| Self-perceived stress-related symptoms, cognitive |  |  |  |  |  |  |  |  |  |  |  | |  |  |
| No  (N=43) | 30 (96.8) | 1 (3.2) | **.04** | 33 (91.7) | 3 (8.3) | .17 | 32 (88.9) | 4 (11.1) | | .29 | 30 (83.3) | | 6 (16.7) | .28 |
| Yes  (N=38) | 28 (80) | 7 (20) |  | 31 (83.8) | 6 (16.2) |  | 33 (89.2) | 4 (10.8) | |  | 27 (73) | | 10 (27) |  |
