# Supplementary material for: Long-term neuropsychiatric and neuropsychological impact of the pandemic in Italian COVID-19 family clusters, including children and parents
Source: PLoS One. 2025 Apr 24;20(4):e0321366. doi: 10.1371/journal.pone.0321366 (PMC12021208; doi:10.1371/journal.pone.0321366)
Supplement: Table S9 — (DOCX) [file pone.0321366.s010.docx]

*Table.S9 - Neuropsychiatric* and neuropsychological symptoms in enrolled parents according to sociodemographic and COVID-19-related factors.

|  | **IES (66/81 = 74.1)** | | | **DASS 21 (73/81 = 90.1)** | | | | | | | | | |
| --- | --- | --- | --- | --- | --- | --- | --- | --- | --- | --- | --- | --- | --- |
|  |  |  |  | ***Depression*** | |  | ***Anxiety*** | |  | ***Stress*** | | |  |
|  | ***<1.1*** | ***≥1.1*** | ***P-value*** | ***≤ 9*** | ***>9*** | ***P-value*** | ***≤ 7*** | ***>7*** | ***P-value*** | ***≤14*** | ***>14*** | | ***P-value*** |
| Overall | 58 (87.9) | 8 (12.1) | - | 64 (87.7) | 9 (12.3) | - | 65 (89) | 8 (11) | - | 57 (78.1) | 16 (21.9) | | - |
| Sex |  |  |  |  |  |  |  |  |  |  |  | |  |
| Female  (N=35) | 26 (89.7) | 3 (10.3) | .28 | 30 (93.8) | 2 (6.2) | .11 | 29 (90.6) | 3 (9.4) | .28 | 25 (78.1) | 7 (21.9) | | .99 |
| Male  (N=44) | 32 (86.5) | 5 (13.5) |  | 34 (82.9) | 7 (17.1) |  | 36 (87.8) | 5 (12.2) |  | 32 (78) | 9 (22) | |  |
|  |  | |  |  | |  |  | |  |  | | |  |
| Non-psychiatric underlying conditions |  |  |  |  |  |  |  |  |  |  |  | |  |
| No  (N=70) | 52 (88.1) | 7 (11.9) | .42 | 59 (88.1) | 8 (11.9) | .40 | 60 (89.6) | 7 (10.4) | .39 | 53 (79.1) | 14 (20.9) | | .39 |
| Yes  (N=9) | 6 (85.7) | 1 (13.3) |  | 5 (88.3) | 1 (16.7) |  | 5 (83.3) | 1 (16.7) |  | 4 (66.7) | 2 (33.3) | |  |
|  |  | |  |  | |  |  | |  |  | | |  |
| Familiarity for neuropsychiatric conditions |  |  |  |  |  |  |  |  |  |  |  | |  |
| No  (N=42) | 43 (93.5) | 3 (6.5) | **.04** | 49 (92.5) | 4 (7.5) | .05 | 48 (90.6) | 5 (9.4) | .24 | 45 (84.9) | 8 (15.1) | | **.02** |
| Yes  (N=37) | 15 (75) | 5 (25) |  | 15 (75) | 5 (25) |  | 17 (85) | 3 (15) |  | 12 (60) | 8 (40) | |  |
|  |  | |  |  | |  |  | |  |  | | |  |
|  |  |  |  |  |  |  |  | |  |  | |  |  |
| SES |  |  |  |  |  |  |  |  |  |  | |  |  |
| Medium-low  (N=36) | 26 (86.7) | 4 (13.3) | .26 | 31 (93.9) | 2 (6.1) | .14 | 29 (87.9) | 4 (12.1) | .26 | 29 (87.9) | | 4 (12.1) | .14 |
| Medium-high  (N=41) | 31 (91.2) | 3 (8.8) |  | 32 (84.2) | 6 (15.8) |  | 35 (92.1) | 3 (7.9) |  | 28 (73.7) | | 10 (26.3) |  |
| COVID-19 |  |  |  |  |  |  |  |  |  |  | |  |  |
| non-COVID-19 cases  (N=13) | 15 (93.7) | 1 (6.3) | .28 | 19 (95) | 1 (5) | .18 | 20 (100) | 0 (0) | .06 | 17 (85) | | 3 (15) | .18 |
| COVID-19 cases  (N=66) | 43 (86) | 7 (14) |  | 45 (84.9) | 8 (15.1) |  | 45 (84.9) | 8 (15.1) |  | 40 (75.5) | | 13 (24.5) |  |
|  |  | |  |  | |  |  | |  |  | | |  |
| Pandemic wave |  | |  |  | |  |  | |  |  | | |  |
| Parental  (N=19) | 16 (94.1) | 1 (5.9) | **.01** | 21 (100) | 0 (0) | **<.01** | 20 (95.2) | 1 (4.8) | .06 | 18 (85.7) | | 3 (14.3) | .30 |
| Delta  (N=34) | 24 (96) | 1 (4) |  | 24 (88.9) | 3 (11.1) |  | 23 (85.2) | 4 (14.8) |  | 22 (81.5) | | 5 (18.5) |  |
| Omicron  (N=26) | 18 (75) | 6 (25) |  | 19 (76) | 6 (24) |  | 22 (88) | 3 (12) |  | 17 (68) | | 8 (32) |  |
|  |  | |  |  | |  |  | |  |  | | |  |
| COVID-19 symptoms^a^ |  |  |  |  |  |  |  |  |  |  | |  |  |
| Asymptomatic  (N=21) | 18 (94.7) | 1 (5.3) | .21 | 22 (95.6) | 1 (4.4) | .12 | 23 (100) | 0 (0) | **.04** | 19 (82.6) | | 4 (17.4) | .53 |
| Symptomatic  (N=58) | 40 (85.1) | 7 (14.9) |  | 42 (84) | 8 (16) |  | 42 (84) | 8 (16) |  | 38 (76) | | 12 (24) |  |
|  |  | |  |  | |  |  | |  |  | | |  |
| Duration of home isolation |  |  |  |  |  |  |  |  |  |  | |  |  |
| <3  (N=31) | 20 (90.9) | 2 (9.1) | .28 | 23 (92) | 2 (8) | .34 | 21 (84) | 4 (16) | .18 | 20 (80) | | 5 (20) | .77 |
| ≥ 3  (N=48) | 38 (86.4) | 6 (13.6) |  | 41 (85.4) | 7 (14.6) |  | 44 (91.7) | 4 (8.3) |  | 37 (77.1) | | 11 (22.9) |  |
