# Supplementary material for: Long-term neuropsychiatric and neuropsychological impact of the pandemic in Italian COVID-19 family clusters, including children and parents
Source: PLoS One. 2025 Apr 24;20(4):e0321366. doi: 10.1371/journal.pone.0321366 (PMC12021208; doi:10.1371/journal.pone.0321366)
Supplement: Table S10 — (DOCX) [file pone.0321366.s011.docx]

*Table.S10 –* Correlation between stress-related symptoms in children and their parents belonging the same COVID-19 family cluster, r (p-value).

|  | **TSCC** | | | **TSCYC** | | |
| --- | --- | --- | --- | --- | --- | --- |
| **IES, r (p-value)** | ***Total*** | ***Daughters*** | ***Sons*** | ***Total*** | ***Daughters*** | ***Sons*** |
| Tot | 0.04 (0.85) | 0.06 (0.85) | 0.03 (0.90) | 0.34 (0.04) | 0.26 (0.30) | 0.39 (0.05) |
| Mother | 0.06 (0.78) | 0.09 (0.78) | 0.09 (0.72) | 0.47 (0.01) | 0.38 (0.14) | 0.48 (0.02) |
| Father | -0.34 (0.26) | -0.60 (0.40) | -0.33 (0.29) | 0.08 (0.72) | -0.10 (0.77) | 0.22 (0.35) |
