# Supplementary material for: Long-term neuropsychiatric and neuropsychological impact of the pandemic in Italian COVID-19 family clusters, including children and parents
Source: PLoS One. 2025 Apr 24;20(4):e0321366. doi: 10.1371/journal.pone.0321366 (PMC12021208; doi:10.1371/journal.pone.0321366)
Supplement: Table S11 — (DOCX) [file pone.0321366.s012.docx]

*Table.S11* – Correlation between emotional-behavioral symptoms in children and their parents belonging the same COVID-19 family cluster, r (p-value).

|  | CBCL, children aged 1.5-5 years | | | CBCL, children aged 6-18 years | | |
| --- | --- | --- | --- | --- | --- | --- |
|  | Total | Internalizing | Externalizing | Total | Internalizing | Externalizing |
| DASS- Depression | 0.45 (0.06) | 0.31 (0.22) | 0.08 (0.75) | 0.29 (0.10) | 0.38 (0.03) | 0.14 (0.42) |
| Mother | 0.64 (<.01) | 0.38 (0.13) | 0.36 (0.16) | 0.27 (0.14) | 0.29 (0.12) | 0.15 (0.43) |
| Father | 0.11 (0.68) | 0.11 (0.67) | -0.25 (0.34) | 0.34 (0.12) | 0.54 (0.01) | 0.17 (0.46) |
| DASS- Anxiety | 0.33 (0.18) | 0.56 (0.02) | 0.23 (0.36) | 0.39 (0.02) | 0.49 (<.01) | 0.28 (0.11) |
| Mother | 0.47 (0.06) | 0.51 (0.04) | 0.29 (0.26) | 0.28 (0.13) | 0.36 (0.05) | 0.14 (0.46) |
| Father | 0.30 (0.25) | 0.51 (0.04) | 0.10 (0.69) | 0.49 (0.02) | 0.60 (<.01) | 0.36 (0.10) |
| DASS-Stress | 0.56 (0.02) | 0.28 (0.26) | 0.23 (0.37) | 0.32 (0.07) | 0.42 (0.01) | 0.24 (0.18) |
| Mother | 0.56 (0.02) | 0.09 (0.72) | 0.52 (0.03) | 0.30 (0.10) | 0.35 (0.06) | 0.30 (0.10) |
| Father | 0.52 (0.03) | 0.44 (0.08) | -0.01 (0.97) | 0.34 (0.13) | 0.49 (0.02) | 0.16 (0.49) |
