# Supplementary material for: Long-term neuropsychiatric and neuropsychological impact of the pandemic in Italian COVID-19 family clusters, including children and parents
Source: PLoS One. 2025 Apr 24;20(4):e0321366. doi: 10.1371/journal.pone.0321366 (PMC12021208; doi:10.1371/journal.pone.0321366)
Supplement: Attachment — Submitted filename: Response to Reviewers.docx [file pone.0321366.s015.docx]

Padua, June 4, 2024

PONE-D-23-36142

Neuropsychiatric and neuropsychological impact of the pandemic in Italian COVID-19 family clusters, including children and parents.

Dear Editor and Reviewers of the *PLOS ONE* Journal,

We thank you for your interest and comments on our manuscript.

We tried to answer the queries at our best. Enclosed you find our replies.

We are pleased to resubmit our manuscript after revision, according to the Academic Editor and Reviewer’s comments.

Costanza Di Chiara, *on behalf of the all authors*

**Editorial comments**

1. Please ensure that your manuscript meets PLOS ONE's style requirements, including those for file naming. The PLOS ONE style templates can be found at

<https://journals.plos.org/plosone/s/file?id=wjVg/PLOSOne_formatting_sample_main_body.pdf> and

<https://journals.plos.org/plosone/s/file?id=ba62/PLOSOne_formatting_sample_title_authors_affiliations.pdf>

**Reply**: We have reviewed our manuscript style following the PLOS ONE author guidelines you provided.

2. We note that the grant information you provided in the ‘Funding Information’ and ‘Financial Disclosure’ sections do not match.

When you resubmit, please ensure that you provide the correct grant numbers for the awards you received for your study in the ‘Funding Information’ section.

**Reply**: We apologize for the error. We confirm that no funding have been received for this manuscript.

3. In the online submission form, you indicated that [The clinical data of the current manuscript are available from the corresponding author upon reasonable request.].

All PLOS journals now require all data underlying the findings described in their manuscript to be freely available to other researchers, either 1. In a public repository, 2. Within the manuscript itself, or 3. Uploaded as supplementary information.

This policy applies to all data except where public deposition would breach compliance with the protocol approved by your research ethics board. If your data cannot be made publicly available for ethical or legal reasons (e.g., public availability would compromise patient privacy), please explain your reasons on resubmission and your exemption request will be escalated for approval.

**Reply**: We confirm that clinical data associated with our study are accessible solely upon reasonable request from the corresponding author. This is in line with the ethical considerations and requirements set forth by our institutional review board and with the informed consent that has been signed by participants/caregivers. The data were collected and stored in a pseudo-anonymized format at the Department for Child and Women's Health of the University of Padova, which is the corresponding author's affiliation. We employed the REDCAP© platform, developed by Vanderbilt University, Tennessee, for this purpose. Upon receiving a motivated request, the corresponding author will provide interested parties with anonymized data. Indeed, to enhance patient privacy, dates of birth will be converted into age ranges before the data are shared.

4. review your reference list to ensure that it is complete and correct. If you have cited papers that have been retracted, please include the rationale for doing so in the manuscript text, or remove these references and replace them with relevant current references. Any changes to the reference list should be mentioned in the rebuttal letter that accompanies your revised manuscript. If you need to cite a retracted article, indicate the article’s retracted status in the References list and also include a citation and full reference for the retraction notice.

**Reply**: The reference list has been thoroughly reviewed and updated to incorporate the latest reference, #21. Additionally, the citation style has been carefully reviewed to ensure adherence to the authors' guidelines.

**Reply to Reviewer**

We have summarized here the amendments made to the manuscript based on the reviewer's comments.

***Introduction***:

1. In "introduction" section, the authors cited that there is in the literature a gap regarding the prevalence and nature of neuropsychiatric and neuropsychological sequelae following COVID-19 in children and adolescents and, only few studies have reported this topic. It is noteworthy that also studies on Covid 19-impact have been conducted in a specific pediatric population, such as children and adolescents with neuropsychiatric disorders (Operto et al). The authors found a statistically significant increase of internalizing problems, anxiety, depression and sleep problems in analyzed sample, with small differences between ages, and high levels of parental stress. These studies confirm that social isolation and restrictions related to lockdown, and the onset of a new infectious disease have had a significant impact on children’s well-being and their families.

In this context, this manuscript is placed, focusing on the prevalence and nature of neuropsychiatric and neuropsychological sequelae following Covid-19 exposure in families and expanding the research on this topic.

**Reply**: We thank the Reviewer for their comment. As suggested, we have included a sentence in the main text highlighting the existence of evidence on the impact of COVID-19 and social restrictions on the well-being of parents and the correlation of parental and familiar stress with the emotional/behavioural worsening of children and adolescents with underlying neuropsychiatric disorders. We have incorporated reference #21 [Operto FF, Scaffidi Abbate C, Piscitelli FT, et al. Adolescents with Neuropsychiatric Disorders during the COVID-19 Pandemic: Focus on Emotional Well-Being and Parental Stress. Healthcare (Basel). 2022;10(12):2368. Published 2022 Nov 25. doi:10.3390/healthcare10122368] into the main text accordingly.

**Changes:** *lines 111-114*: The social restrictions imposed during the pandemic have notably disrupted daily family routines, increasing stress in children and parents. This stress potentially leads to the deterioration of children's mental health as well as exacerbates emotional and behavioral symptoms in adolescents with underlying neuropsychiatric disorders.^21^

***Results section***:

1. The authors cited that participants who lacked analytical evidence of SARS-CoV-2 infection were designated as "nonCOVID-19" cases. Why they were included as asymptomatic ("severity of COVID-19" section) in Table 1?

**Reply**: We appreciate the reviewer's feedback. Initially, in Table 1, we included non-COVID-19 cases labeled as asymptomatic, confirmed by negative SARS-CoV-2 IgG test results at enrollment, to highlight their lack of symptoms during their COVID-19 family cluster. However, upon reflection, we acknowledge that this detail is redundant considering its clear explanation in the text. Consequently, we have removed the count of asymptomatic non-COVID cases from Table 1.

**Changes:** Please refer to Table 1 in the main text. The counts of asymptomatic non-COVID-19 cases have been removed from the 'overall', 'children', and 'parents' columns.

2. In table 2 (and tables in supplemental materials), the statistically significant p-value should be highlighted in bold or with other mark, to make the correlation clearer

**Reply**: We appreciate the reviewer's suggestion. Statistically significant p-values have been highlighted in bold in all main and supplemental tables.

**Changes:** please refer to the tables in both the main text and the supplemental material documents.

3. The figures are not clear enough to display correctly due to their blurriness.

**Reply:** We appreciate the Reviewer for bringing this matter to our attention, and we apologize for the poor quality of the uploaded graph. We have saved them using a different method, and they should now be legible.

**Change:** Please refer to the uploaded graphs for the necessary changes.
