# Supplementary material for: Long-term neuropsychiatric and neuropsychological impact of the pandemic in Italian COVID-19 family clusters, including children and parents
Source: PLoS One. 2025 Apr 24;20(4):e0321366. doi: 10.1371/journal.pone.0321366 (PMC12021208; doi:10.1371/journal.pone.0321366)
Supplement: Attachment — Submitted filename: PONE-review.doc [file pone.0321366.s014.doc]

This study investigated the long-term effetct of pandemic COVD-19 on neuropsychiatric and neuropsychological impact in 46 Italian families, including children and parents, attending the COVID-19 Follow-up Clinic at the Department for Women’s and Children’s Health,

Padua (Italy) from December 2021 to November 2022.

A total of 81 parents and 77 children were asessed for Self-perceived stress-related,

emotional-behavioral, and post-traumatic stress (PTSD-related) symptoms. Overall,125 (79%) and 33 (21%) participants were classified as COVID-19 cases and non-COVID-19 cases, respectively.

The time interval between the COVID-19 family outbreak and the neuropsychiatric and psychological assessment

was ≤4 months for 89 (56.3%) participants and >4 months for 69 (43.8%) participants.

Authors conclude that while a higher prevalence of self-perceived stress-related symptoms

was found in COVID-19 cases compared to non-COVID-19 cases (p=.01), no

differences were observed for emotional-behavioral and PTSD-related symptoms

between the two groups.

The study is interesting and may stir interest in the reader. Nonethelss there are major criticisms about this study authors should deal with:

- Abstract: it is not enough clear if this data actually assess the long term effects ; why long-term if the data were gathered less than four months in many families; and how long the follow up was after 4 months?
- The term long term should be stressed also in the title
- It is not clear which were the mild of moderate/severe symptoms these patients developed. These shoudl be clarified in results and discussion
- It is clear that this study is too much limited by a small intrafamilies no covid group and by the absence of a control group of families with no-COVID and isolation only in the same geographic area.
- It is not as well clear in the text the effect of the interval from COVID onset and neuropsichological/psychiatric assessement: was a longer interval (> 4 months) associated with a stress reduction?
- Authors should try to comment on higher non verbal memory functioning and precessing speed in 5% of children from COVID family clusters due to Omicron vs Delta virus.
